# Capsid coding sequences of foot-and-mouth disease viruses are determinants of pathogenicity in pigs

**DOI:** 10.1186/1297-9716-43-46

**Published:** 2012-05-24

**Authors:** Louise Lohse, Terry Jackson, Anette Bøtner, Graham J Belsham

**Affiliations:** 1 National Veterinary Institute, Technical University of Denmark, Lindholm, Kalvehave, DK-4771, Denmark; 2 Institute for Animal Health, Pirbright, Woking, Surrey, GU24 ONF, United Kingdom

## Abstract

The surface exposed capsid proteins, VP1, VP2 and VP3, of foot-and-mouth disease virus (FMDV) determine its antigenicity and the ability of the virus to interact with host-cell receptors. Hence, modification of these structural proteins may alter the properties of the virus.

In the present study we compared the pathogenicity of different FMDVs in young pigs. In total 32 pigs, 7-weeks-old, were exposed to virus, either by direct inoculation or through contact with inoculated pigs, using cell culture adapted (O1K B64), chimeric (O1K/A-TUR and O1K/O-UKG) or field strain (O-UKG/34/2001) viruses. The O1K B64 virus and the two chimeric viruses are identical to each other except for the capsid coding region.

Animals exposed to O1K B64 did not exhibit signs of disease, while pigs exposed to each of the other viruses showed typical clinical signs of foot-and-mouth disease (FMD). All pigs infected with the O1K/O-UKG chimera or the field strain (O-UKG/34/2001) developed fulminant disease. Furthermore, 3 of 4 in-contact pigs exposed to the O1K/O-UKG virus died in the acute phase of infection, likely from myocardial infection. However, in the group exposed to the O1K/A-TUR chimeric virus, only 1 pig showed symptoms of disease within the time frame of the experiment (10 days). All pigs that developed clinical disease showed a high level of viral RNA in serum and infected pigs that survived the acute phase of infection developed a serotype specific antibody response. It is concluded that the capsid coding sequences are determinants of FMDV pathogenicity in pigs.

## Introduction

Foot-and-mouth disease (FMD) is one of the world’s most economically important infectious diseases of farm animals including cattle, pigs and sheep. The aetiological agent of FMD is foot-and-mouth disease virus (FMDV) which is the prototype *Aphthovirus* within the family *Picornaviridae*. The virus can infect about 70 different wild life species and can spread rapidly causing high morbidity but only low mortality except in young animals [1]. The severity of the disease varies between hosts with pigs and cattle exhibiting clear clinical signs while infection in sheep is often difficult to detect by clinical observation. The disease in cattle and pigs follows a rapid time course and causes a rise in body temperature and the development of vesicular lesions in and around the mouth and on the feet. Infected animals display varying degrees of salivation, inappetence and lameness according to the severity of lesions. The clinical course of the infection usually subsides within 7–14 days and is accompanied by a rapid generation of neutralizing antibodies within the serum. However, many animals can subsequently “carry” the virus in the oropharynx for a prolonged period; this “carrier state” can be maintained for several months (in sheep) or years (in cattle and buffalo). In general it is considered that pigs do not become carriers [1] although there is some evidence to the contrary [2-4].

FMDV exists in 7 distinct serotypes, O, A, C, SAT1, SAT2, SAT3 and Asia-1. Each virus particle contains a positive sense single-stranded RNA genome of about 8.4 kb enclosed within a protein shell consisting of 60 copies each of the 4 capsid proteins 1A (VP4), 1B (VP2), 1C (VP3) and 1D (VP1) [5]. VP1, VP2 and VP3 are exposed on the outer surface of the virus particle and hence they determine both the antigenicity of the virus and its ability to interact with specific receptors on cells.

The major cellular receptor for FMDV is believed to be the integrin αvβ6 which is expressed on epithelial cells [6-8] but other RGD-binding integrins, (αvβ8, αvβ1 and αvβ3), have also been shown to function as receptors for FMDV in cell culture [8-11]. Cell culture adapted serotype O viruses can also bind to cells via heparan sulfate (HS) and can use this interaction to initiate infection. The ability to bind to HS is associated with specific amino acid substitutions (including His56 to Arg56 in VP3) on the surface of the virus [12-14]. However, the HS binding phenotype has been linked to attenuation of serotype O virus in cattle [14,15].

Different strains of FMDV can vary greatly in their pathogenicity within different host species. For instance, some virus strains (e.g. O/Taiwan 1997) cause disease in pigs but not in cattle (i.e. the porcinophilic strains) [16]. Some of these viruses (namely O/Taiwan 1997) have been shown to contain a deletion within the coding region for the 3A protein [17,18]; however, other porcinophilic strains from Korea (O/SKR/AS/2002) had an intact 3A coding region [19].

Full-length infectious cDNAs corresponding to the FMDV genome have been produced for serotype O [20-22] and serotype A [23,24] viruses. These have permitted the construction of chimeric full-length infectious FMDV cDNAs in which the capsid coding sequences of one virus has been replaced with the equivalent coding region of a different virus. Viable chimeric viruses have been rescued from these cDNAs and been shown to display the antigenicity of the introduced capsid sequences [14,15,25,26].

Previously we have replaced the coding region for the surface exposed capsid proteins (VP1, VP2 and VP3) of an infectious cDNA derived from a cell culture adapted, HS-binding, strain of O1 Kaufbeuren (O1K B64) (pT7S3, see [22]) with the corresponding region of field strains of serotype O (O/UKG/34/2001) or serotype A (A-Tur/06) viruses [15]. Thus the chimeric viruses obtained (O1K/O-UKG and O1K/A-Tur) and the parental O1K B64 are identical to each other except for the capsid coding region. In the previous study, these chimeric viruses were shown to be pathogenic in cattle whereas the parental O1K B64 strain is completely attenuated in this host [15]. Thus the production of chimeric viruses can be a useful tool for identifying determinants of pathogenicity.

Since the pathogenicity of FMDVs can vary between hosts we have now determined the virulence of these chimeric viruses and their parent (O1K B64) within pigs as well. For comparison the O/UKG/34/2001 field virus was tested in parallel. This virus produces a relatively mild form of the disease in cattle [27] but has also been well characterized within experimental studies in pigs in which it causes quite severe disease [28,29].

Here we report that the rescued cell culture adapted O1K B64 virus is also highly attenuated in pigs. However, the two chimeric virus derivatives, O1K/O-UKG and O1K/A-TUR, were able to cause typical clinical signs of FMD in inoculated pigs albeit with different efficiencies. Some young pigs infected by contact exposure to the O1K/O-UKG chimera died suddenly during the acute phase of infection. These studies show that the capsid coding sequences are determinants of FMDV pathogenicity in pigs.

## Material and methods

### Animal experiments and samples

Experimental procedures and animal management protocols were carried out in accordance with the requirements of the Danish Animal Experimentation Inspectorate, license no. 2008/561-1541. All pigs were housed in the high containment BSL3+ experimental facilities at the National Veterinary Institute, Lindholm.

In total, 32 weaner pigs (7 weeks of age) were obtained from a conventional Danish swine herd with specific pathogen free (SPF) status, including freedom from enzootic pneumoniae, atrophic rhinitis, swine dysentery, porcine reproductive and respiratory syndrome and most serotypes of pleuropneumoniae. All pigs were found to be healthy by veterinary inspection on arrival at the Institute, 1 week before the start of the experiment. All pigs were fed a commercial diet for weaning pigs and water was provided *ad libitum*. Straw was used for bedding.

Pigs were randomly divided into 4 groups of 8 pigs and the individual groups were kept in separate pens with no physical contact between them. Groups 1 and 2, in one stable, shared the same air space while Groups 3 and 4, in another stable, also shared air space with each other. Four pigs in each group were inoculated with the different FMDVs: Group 1 was inoculated with the cell culture adapted B64 strain of the O1 Kaufbeuren virus (O1K B64); Group 2, was inoculated with the O1K/A-TUR chimeric virus [15], a derivative of O1K B64 containing the surface exposed capsid proteins (VP1, VP2 and VP3) from A/Turkey 2/2006; Group 3 was inoculated with a second chimeric virus (O1K/O-UKG) [15] also derived from O1K B64 and containing VP1, VP2 and VP3 from O UKG/34/2001 and Group 4 was inoculated with the field strain O UKG/34/2001, as used previously at Lindholm, in cattle by Stenfeldt et al. [27]. Inoculation was performed in the heel bulb, each pig receiving approximately 10^5^ TCID_50_ in a volume of 0.3 mL. All inoculated pigs were anaesthetized during the process. The remaining four pigs in each group were left as in-contact animals.

On each subsequent day, all pigs were examined for clinical signs of FMD, to generate a clinical score, and rectal body temperatures were recorded. Clinical scores (CS) were assigned based on a semi-quantitative scoring system with each animal being assessed on 4 FMD-relevant parameters (general well-being, appetite, lameness and the presence of lesions on the mouth/nose/tongue and feet). Body weight was recorded on arrival at the Institute and at the termination of the experiment. Blood samples were collected from the jugular vein on the following days post infection (dpi) 0, 1, 2, 3, 4, 5, 7 and 10 for inoculated pigs and on dpi 0, 4, 5, 6, 7, 8, 9 and 10 for contact pigs. Oral swab samples were collected at the same times. Pigs, which developed significant lesions, were treated with analgesics. Pigs that displayed clinical symptoms which reached the humane endpoint criteria during the course of the experiment, were euthanised when necessary and, after 10 days, surviving pigs were euthanised by intravenous injection of sodium pentobarbital. All pigs were autopsied and relevant tissues were collected for further examination.

### Quantification of anti-FMDV antibodies using a solid phase blocking ELISA

Serum samples were screened for antibodies to FMDV in a solid phase blocking ELISA [30,31]. In brief, microtitre plates were coated with guinea-pig immune serum raised against FMDV serotype O or serotype A, as appropriate, before addition of the respective inactivated FMDV antigen. Test serum samples were then added and incubated overnight, the plates were washed and then rabbit anti-FMDV serotype O or A serum was added. Bound rabbit antibodies were detected with horseradish-peroxidase conjugated pig anti-rabbit IgG (Dakopatts P0217, DakoCytomation, Denmark A/S). All serum samples were initially screened at a dilution of 1:5 and positive samples were further analyzed in a two-fold titration, starting at a dilution of 1:10 to allow determination of antibody titers.

### Quantification of viral RNA using a quantitative real time RT-PCR

FMDV RNA in serum, oral swabs and heart tissue was determined using a quantitative real-time RT-PCR assay [32] which is targeted to the 5'-untranslated region of the viral RNA. In brief, total RNA was extracted from 200 μL of sample using a MagNa Pure LC Total Nucleic Acid Isolation Kit (Roche) according to the manufacturer′s instructions in an automated RNA extraction system. After extraction, each sample was eluted in 50 μL Elution Buffer. cDNA was made using 6 μL of extracted RNA in a total volume of 15 μL, using a TaqMan RT kit mastermix with random hexamer primers (Applied Biosystems) at 48°C for 45 min and 95°C for 5 min. Seven μL of cDNA was mixed with 18 μL of 2× TaqMan universal PCR mastermix (Applied Biosystems) containing 22.5 pmol of each primer and 7.5 pmol of fluorescently labeled probe. PCR amplification was carried out for 50 cycles in a Thermal cycler (Mx3005P, Stratagene) and analysed using MxPro qPCR software (Stratagene). Standard curves were produced using 10-fold dilutions (10^0^-10^7^) of the synthesis control (an RNA transcript from a plasmid containing part of the 5′ untranslated region of FMDV strain O/UKG/34/2001).

### Sequencing of viral RNA

To determine if cross contamination between groups sharing the same air space had occurred, sequencing of viral RNA isolated from serum and heart tissue from pigs infected with the O1K/O-UKG (Group 3) and O-UKG/34/2001 (Group 4) viruses was performed. In brief, random primed cDNA was made, as described previously [33] and PCR products corresponding to most of the VP1 (ca. 520 bp) and to part of the VP4/VP2 coding regions (ca. 820 bp) were amplified and sequenced in both directions. Sequences generated were compared to those known for O1Kaufbeuren [GenBank: X00871] and O/UKG/35/2001 [GenBank: AJ539141] using BLASTN.

### Statistics

Data analysis was performed using GraphPad In Stat version 3.00 (GraphPad Software, San Diego, CA, USA). One way ANOVA with post test was selected for comparison of mean body weights between groups.

## Results

### Clinical findings

**Figure 1:**
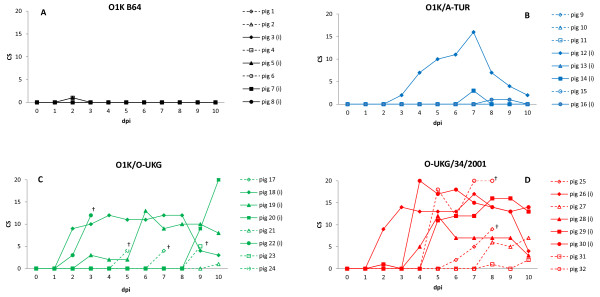
**Clinical scores (CS) for individual pigs.** A CS was assigned to each animal based on 4 FMD-relevant parameters: 1) Well-being: 0 = normal, 1 = depressed; 2) appetite: 0 = normal, 1 = suppressed; 3) mobility: 0 = free movement, 1 = lame, 2 = recumbent; 4) lesions on mouth/nose/tongue/feet (all 4 feet individually scored): 0 = no lesion, 1 = elevated temperature in local area/congestion/healing vesicle, 2 = vesicle, 3 = ruptured vesicle/severe lesion, in total giving a score between 0 and 25. Inoculated pigs = filled symbols and solid lines, in-contact pigs = open symbols and dotted lines. † indicates death or euthanasia for welfare reasons. Panel A: Group 1 (O1K B64), panel B: Group 2 (O1K/A-TUR), panel C: Group 3 (O1K/O-UKG), panel D: Group 4 (O-UKG/34/2001).

Four pigs within each of four groups of pigs were inoculated with different FMDVs and, within each group, four other pigs were left as “in-contact” pigs (see Materials and methods). Clinical signs of FMD within the pigs varied between the groups exposed to the cell culture adapted O1KB64, chimeric (O1K/O-UKG and O1K/A-TUR) or field strain (O-UKG/34/2001) viruses. Clinical scores for the individual pigs within the 4 groups are shown in Figure 1. The results show that pigs inoculated with the cell culture adapted virus, O1K B64, or exposed to it by direct contact with the inoculated pigs (Group 1), did not develop any clinical signs of FMD. In contrast, pigs in each of the 3 other groups developed moderate or severe symptoms of FMD, including vesicular lesions in the oral cavity and also on the feet (mainly along the coronary band and in the heel bulb area) which resulted in lameness. Pigs affected by disease typically showed slight depression and loss of appetite, while only sporadic temperature increases in individual pigs were observed (data not shown).

In more detail, only one pig in Group 2 (pig 12) which was inoculated with the chimeric serotype A virus (O1K/A-TUR) developed severe disease with a maximum clinical score of 16 (see Figure 1b). Clinical disease was not observed in the contact pigs in the 10 day experiment (but see below). However, all pigs (in Group 3) exposed to the O1K/O-UKG chimeric virus (inoculated and contacts) developed FMD, including vesicles in the oral cavity and on the feet, with clinical scores of up to 20 (Figure 1c). In this Group, one inoculated pig (id no. 22), and 3 of the 4 contact pigs (id nos. 17, 23, 24) were found dead in the pen only one day after the first appearance of clinical signs. The remaining contact pig (id no. 21) did not develop vesicular lesions indicative of FMD, however, at dpi 10 (the day of termination of the experiment) lameness was recorded. Pigs in group 4 exposed by inoculation or through contact to the O-UKG/34/2001 field strain all developed apparent clinical disease with clinical scores of between 12 and 20 (Figure 1d). Two contact pigs in this group (id nos. 25 and 32) were euthanised on dpi 8 for animal welfare reasons, as administration of analgesics did not appear to relieve their pain.

Growth rate, defined as the weight difference between the start and termination of the experiment was calculated for the individual pigs. Mean weight difference for the 4 groups were compared and no significant difference between groups were found (*p* > 0.05; data not shown). Weight gain for pigs included in the experiment for the full time period was on average 2.3 kg. Pigs, which died or were euthanised before the conclusion of the experiment, were not weighed, however, by visual inspection at the post mortem (PM) examination these pigs appeared to be of fair condition.

### Autopsy

PM examination revealed mostly lesions or healed lesions on the feet and in the oral cavity typical of FMD. In one pig (id no. 11), however, light grey areas on the left ventricle, compatible with myocardial degeneration in the heart, was observed. This finding was not related to any signs of clinical illness, but this pig showed rising levels of viral RNA in serum (see below) on the last days before termination of the experiment and could therefore have been at risk of acute death from myocardial infarction, if the experiment had been prolonged.

### Virus detection in serum

**Figure 2:**
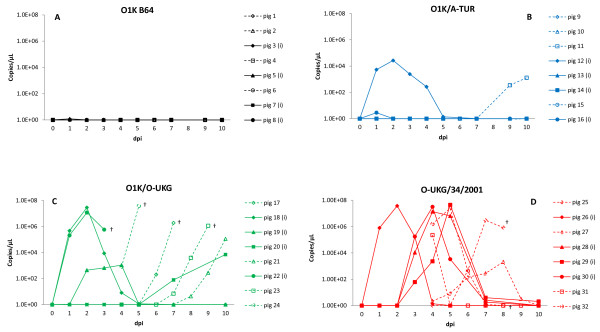
**Viral RNA in serum, from individual pigs, measured by quantitative real-time RT-PCR expressed as copies of FMDV genome/μL of serum.** Inoculated pigs = filled symbols and solid lines, in-contact pigs = open symbols and dotted lines. Blood samples were collected on dpi 0, 1, 2, 3, 4, 5, 7 and 10 for inoculated pigs and on dpi 0, 4, 5, 6, 7, 8, 9 and 10 for contact pigs. † indicates death or euthanasia for welfare reasons. Panel A: Group 1 (O1K B64), panel B: Group 2 (O1K/A-TUR), panel C: Group 3 (O1K/O-UKG), panel D: Group 4 (O-UKG/34/2001).

The level of FMDV RNA was determined in serum samples collected at different time points throughout the course of the experiment (Figure 2). Inoculated pigs, which showed clinical signs of disease, had high levels of FMDV RNA (up to 10^7^ genome copies/μL) from dpi 1, reflecting rapid replication and systemic dissemination of the virus. For all pigs with clinical symptoms of FMD (inoculated and contacts) viral RNA was detected in serum for a period of 4–6 days. Typically, viral RNA was detected in serum for one day before clinical signs appeared. The results from each Group will be considered below in more detail.

#### O1K B64 (Group 1)

No clinical signs of FMD were observed in this group but one out of 4 pigs (id no. 5) inoculated with the cell culture adapted virus had detectable but very low amounts of viral RNA (1.2 genome copies/μL) in serum for one day only (Figure 2a). All other pigs in this Group (inoculated and contacts) had no detectable viral RNA in their serum. Thus there was very limited evidence for any replication and no sign of any transmission of the virus from inoculated to contact pigs.

#### O1K/A-TUR (Group 2)

In the pigs inoculated with the O1K/A-TUR chimeric virus, one inoculated pig (id no. 12), which showed clinical disease, had relatively high levels of viral RNA in serum from dpi 1–5 (up to 10^4^ genome copies/μL). One other inoculated pig (id no. 16) had a very low level of viral RNA in serum (2.84 genome copies/μL) for one day after inoculation, however, no clear clinical symptoms were ever observed in this pig. In addition, one contact pig (id no. 11) had rising levels (up to 10^3^ genome copies/μL) of FMDV RNA, starting at dpi 9 (Figure 2b). However, as the experiment was terminated on dpi 10, the development of the infection in this pig could not be followed but, as indicated above, this pig did exhibit signs of myocardial degeneration in the heart at autopsy.

#### O1K/O-UKG (Group 3)

Pigs inoculated with the O1K/O-UKG chimeric virus, all showed clinical signs of FMD and had viral RNA detectable in serum for up to five days. Three out of 4 inoculated pigs (id nos. 18, 19, 22) were positive from dpi 1, while the fourth (id no. 20) became positive during the same time period as the contacts, i.e. from dpi 4 and onwards. The sudden deaths of one of the inoculated pigs and three of the four in-contact pigs clearly precluded detailed analysis of the progression of the infection in these animals, however, all these 4 pigs had high levels of FMDV RNA (10^5^ to 10^7^ genome copies/μL) in their serum on the day before they died (Figure 2c).

#### O-UKG/34/2001 (Group 4)

In the Group infected with the field strain O-UKG/34/2001, one inoculated pig (id no. 26) was viral RNA positive in serum on dpi 1, while the remaining 3 inoculated pigs (id nos. 28, 29, 30) were positive from dpi 3 (all up to 10^7^ genome copies/μL), see Figure 2d. Contact pigs in this group were identified as positive for virus RNA from dpi 4 and onwards, but could have been positive at an earlier time point since the first serum samples from the contact pigs were only taken at dpi 4 after the null-sample (for animal welfare reasons). Therefore we do not know the true time point for the first appearance of virus in the blood of contact pigs tested positive on this day.

Whether a delayed infection or transmission of virus by contact was the case for some of the inoculated pigs (id nos. 28, 29, 30 in Group 4 and also id no. 20 in Group 3) that responded “late” (i.e. 3–7 days after inoculation) in this experiment is not clear.

### Detection of FMDV RNA in oral swab samples

**Figure 3:**
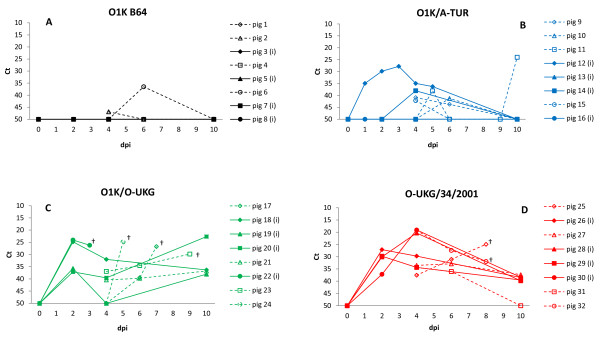
**Viral RNA in oral swab samples, from individual pigs, measured by quantitative real-time RT-PCR and expressed as threshold cycle (Ct) values (note when no Ct value was obtained by 50 cycles then a value of 50 was used).** Inoculated pigs = filled symbols and solid lines, in-contact pigs = open symbols and dotted lines. Swab samples were collected on dpi 0, 1, 2, 3, 4, 5, 7 and 10 for inoculated pigs and on dpi 0, 4, 5, 6, 7, 8, 9 and 10 for contact pigs and selected samples were assayed. † indicates death or euthanasia for welfare reasons. Panel A: Group 1 (O1K B64), panel B: Group 2 (O1K/A-TUR), panel C: Group 3 (O1K/O-UKG), panel D: Group 4 (O-UKG/34/2001).

The level of FMDV RNA in oral swab samples showed a pattern consistent with the detection of viral RNA in serum, but at lower levels (c.f. Figure 2 and Figure 3). The Ct-values indicated about a 100-fold lower virus load in oral swab samples compared to serum and we believe that a more precise quantification of the virus in swab samples from the mouth is not possible (e.g. due to variable level of salivation etc.). However, detectable viral RNA in oral swab samples was typically observed over a longer time span than in serum, often 6–9 days. In fact, for the pigs containing the highest levels of virus (pigs in Groups 3 and 4) the oral swab samples still contained detectable levels of FMDV RNA at the day of termination (dpi 10). Furthermore, FMDV RNA was found at low levels sporadically in the oral swab samples of several pigs not going through a systemic FMDV infection in the groups inoculated with virus of low virulence, i.e. O1K B64 and O1K/A-TUR infected groups (Figure 3).

### FMDV RNA in heart tissue

Due to the sudden death of several pigs from Group 3 (exposed to the O1K/O-UKG chimera), the presence of FMDV RNA was assayed in heart tissue from pigs infected with this virus and (for comparison) in samples from pigs in Group 4 (exposed to the O-UKG/34/2001 field strain). Based on Ct-values, the pigs could be divided into 3 sets. The first set was comprised of the pigs that died in the acute state of infection, after showing initial clinical symptoms (id nos. 17, 22, 23, 24). These pigs had high levels of FMDV RNA (Ct-values = 17–18) in heart tissue as well as in serum (Ct-values = 14–19). The two other sets included pigs that survived until the end of the experiment. The first of these sets included pigs that developed infection late in the experimental period, i.e. first detectable level of viral RNA in serum at dpi 7–9 (id nos. 20, 21, 25). These pigs were positive for detection of FMDV RNA in heart tissue (Ct = 23–33) and serum (Ct = 17–26), but had a lower load of virus than pigs that died in the acute phase of infection. The second set of pigs that survived until the end of the experiment included pigs that were past or almost past the acute phase of infection (id nos. 18, 19, 26, 27, 28, 29, 30, 31, 32). These pigs were negative or had low levels of virus RNA by RT-PCR analysis for FMDV RNA in both heart tissue and serum at this stage.

### Sequencing of viral RNA isolated from serum and heart tissue

Pigs within Groups 3 (O1K/O-UKG chimera) and 4 (O-UKG/34/2001 field strain) were housed within the same air space although they were well separated (by over 3 m); previous studies have shown that a physical gap of 70 cm [34] is sufficient to prevent spread of virus by aerosol between pigs. However, we wanted to ensure that the clinical results observed reflected the response to the specific virus used for that Group. Thus, RNA was extracted from serum and heart tissue samples and used to amplify, by RT-PCR, fragments corresponding to most of the VP1 coding region (ca. 520 bp), that is common to each of these viruses, and to part of the VP4/VP2 coding region (ca. 820 bp), which includes the junction between the O1K B64 and the O-UKG capsid coding sequence in the chimera. As expected, the VP1 sequences were identical to the O-UKG/35/2001 sequence [GenBank: AJ539141] in each of the 3 samples tested (from pigs with id nos. 23, 24 and 25). However, around the junction between the VP4 and VP2 coding sequences, RNA obtained from pigs with id nos. 23 and 24 (Group 3) matched the expected structure of the O1K/O-UKG chimera while the sequence generated from the pig with id no. 25 sample (Group 4) exactly matched the O-UKG/35/2001 sequence (data not shown). Thus these results indicated that these pigs had indeed been infected with the expected virus.

### Development of FMDV specific antibodies

**Figure 4:**
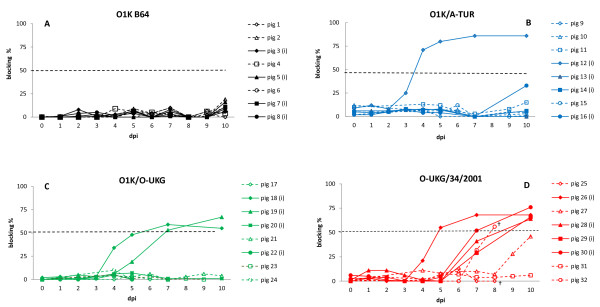
**FMDV serotype specific (O or A) antibody response in serum expressed as blocking percentage derived from solid phase blocking ELISA.** Diagnostic cut-off level is 50% for serotype O and 45% for serotype A. Inoculated pigs = filled symbols and solid lines, in-contact pigs = open symbols and dotted lines. Blood samples were collected on dpi 0, 1, 2, 3, 4, 5, 7 and 10 for inoculated pigs and on dpi 0, 4, 5, 6, 7, 8, 9 and 10 for contact pigs. † indicates death or euthanasia for welfare reasons. Panel A: Group 1 (O1K B64), panel B: Group 2 (O1K/A-TUR), panel C: Group 3 (O1K/O-UKG), panel D: Group 4 (O-UKG/34/2001).

Anti-FMDV specific antibodies were produced in pigs which developed a systemic FMD infection and survived. Three to five days after virus was detected in serum, antibodies with the expected serotype specificity (O or A) could be demonstrated. The diagnostic cut-off level, defined as 45% (for serotype A) or 50% (for serotype O) blocking of the serotype-specific antigen in ELISA, was reached after 5–7 days (Figure 4). Contact pigs which died acutely in the group of O1K/O-UKG infected pigs did not survive long enough to develop such antibodies. No antibodies were detected in animals that did not exhibit signs of clinical disease.

## Discussion

The pathogenicity in young pigs of 4 different, genetically defined, FMDVs has been compared. The properties of the cell culture adapted O1K B64 virus and its chimeric derivatives (O1K/O-UKG and O1K/A-TUR) have been investigated in cattle previously [15] while the O/UKG/34/2001 field virus has been characterized in separate studies in pigs [28,29] and cattle [27].

The cell culture adapted O1K B64 did not cause clinical signs of FMD in pigs as was observed previously in cattle [15]. Viral RNA in serum was only found at low levels within one pig on a single day. It seems that this HS-binding virus either does not reach the blood or else is rapidly cleared from the circulation and thus does not get the opportunity to spread and replicate within the inoculated animals. In contrast, both the O1K/O-UKG and the O1K/A-TUR chimeric viruses, which have capsid proteins from field strains but are otherwise identical to the O1K B64 virus, caused classical symptoms of FMDV infection in pigs, albeit at different levels. The O1K/O-UKG virus caused severe disease in pigs with 100% morbidity and an overall mortality of 50%, indeed for the in-contact pigs mortality was 75%. These results are similar to those seen in cattle [15] except that no mortality was observed in this species. In contrast, the O1K/A-TUR chimera showed much lower disease impact in pigs (only 1 of the 4 inoculated animals showed clinical signs) whereas in cattle 5 of 6 animals in the group exposed to this chimera became infected within a similar experiment [15]. Furthermore, one of the pigs seemed to become infected with this chimeric virus late in the experiment (as judged by the appearance of viral RNA in serum, see Figure 2b) but due to the short time perspective (the experiment was terminated after 10 days) development of clinical disease and possible transmission to pen-mates could not be followed, however, signs of myocardial degeneration were observed in this pig at autopsy. Therefore a higher morbidity rate for the pigs might have been seen if this experiment had been prolonged. Pigs inoculated with the field strain O-UKG/34/2001 demonstrated severe clinical disease and high levels of viral RNA in serum. Clinical signs (lameness, severe vesicular lesions along coronary bands and heel bulb area and to a lesser extent in the mouth region) closely resembled symptoms described in earlier studies [28,29] and levels of viral RNA in serum (peak concentrations of 10^10^ genome copies/mL) were very high (c.f up to 10^9.7^ genomes/mL determined by Quan et al. [28]). This virus strain causes more severe disease in pigs than in cattle [27,29].

A corresponding serotype specific antibody response was developed in all pigs that became systemically infected and then survived through the acute phase, irrespective of which virus or derivative the individual pig was exposed to.

In this study we saw acute deaths in pigs exposed to the O1K/O-UKG chimera. Three out of 4 contact pigs were found dead in the pen shortly after initial clinical signs were recorded. High amounts of viral RNA was found in the heart (myocardium) of these pigs. Fatal FMD is often associated with myocarditis, a phenomenon recognized to occur only in young animals. In these cases macroscopical examination of the heart often reveals a soft, flaccid heart with white or greyish stripes, so-called “tiger heart”. However, there may be no significant macroscopical lesions, but virus can usually be isolated from the myocardium [1]. It seems likely that acute myocarditis was the cause of death in pigs in our study, even though no myocardial degeneration was visually detected in these pigs (id nos. 17, 22, 23, 24), due to the high levels of FMDV RNA detected in the heart of these animals. During the viraemic phase of disease, virus can be found in all organs; therefore organs should be regarded as sites of active viral replication only when they show a higher concentration of virus or viral RNA than found in blood at the same time [1]. In the present study, the level of FMDV RNA in corresponding serum and heart tissue samples was similar (as judged by Ct levels). However, during the RNA preparation procedure (for extraction and RT-PCR) the heart tissue samples were diluted 1:10 for homogenization and therefore the viral load in heart tissue should be regarded as even higher than in serum. This indicates a specific secondary replication of virus in the heart musculature of these pigs and was only observed in pigs that died in the acute phase of infection. It is not clear why the O1K/O-UKG chimeric virus should cause a higher rate of death than the parental field strain (since the level of viral RNA reached in serum was similar) but this may just be a consequence of the relatively small number of animals involved. Interestingly, one contact pig (id no. 11) from Group 2, infected with the other chimeric virus (O1K/A-TUR), showed macroscopic lesions compatible with myocardial degeneration and could have been a candidate for myocardial infarction. Viral RNA in oral swab samples was found at low levels in several pigs which were not going through a systematic FMDV infection, especially in pigs from Group 2 inoculated with the O1K/A-TUR chimera. This probably represents background environmental virus that had been inhaled and trapped in the respiratory tract originating from the single pig which developed clinical disease.

In Groups 3 and 4, the majority of the pigs inoculated with virus (the O1K/O-UKG chimera or the O-UKG/34/2001 field strain, respectively) developed disease on dpi 2–4. However, 2 pigs (id nos. 20 and 29) developed disease at dpi 9 and dpi 5, respectively, i.e. in the same time interval as the in-contacts, which developed disease from dpi 5 and onwards. Whether these 2 inoculated pigs responded late to the inoculated virus or should be regarded as contact infected pigs cannot be determined.

Continuing epidemics of FMDV infection in large parts of the world, remind us of the necessity of still putting effort into FMDV research in order to broaden our knowledge regarding the pathogenicity of this virus and the specific host-virus interactions which underlie this. The chimeric viruses used here provide a useful tool to determine the influence of the capsid proteins alone on the properties of virus and the experiments in the natural host animals are critical for understanding how these factors determine the outcome of virus infection. Since the O1K B64, O1K/O-UKG and O1K/A-TUR viruses are genetically identical except for their capsid protein coding regions, but differ markedly in their ability to induce disease, the clear conclusion from these studies is that these capsid proteins are determinants of FMDV pathogenicity in swine.

## Competing interests

The authors declare that they have no competing interests.

## Authors’ contributions

LL planned and performed the animal experiment and analysed the data. TJ designed and assembled the chimeric FMDV cDNA clones. AB participated in the overall planning of the experiment. GJB rescued the chimeric FMDVs, participated in the overall planning of the animal experiment and analysed the data. LL and GJB wrote the manuscript. All authors commented and approved the final manuscript.
